# Twice evasions of Omicron variants explain the temporal patterns in six Asian and Oceanic countries

**DOI:** 10.1186/s12879-023-07984-9

**Published:** 2023-01-13

**Authors:** Boqiang Chen, Yanji Zhao, Zhen Jin, Daihai He, Huaichen Li

**Affiliations:** 1 Department of Applied Mathematics, Hong Kong Polytechnic University, Hong Kong, China; 2 Complex Systems Research Center, Shanxi University, Taiyuan, China; 3 Department of Respiratory and Critical Care Medicine, Shandong Provincial Hospital Affiliated to Shandong First Medical University, Jinan, China

**Keywords:** COVID-19, The Omicron variant, Epidemic model, Reproduction number, Infection fatality rate

## Abstract

**Background:**

The ongoing coronavirus 2019 (COVID-19) pandemic has emerged and caused multiple pandemic waves in the following six countries: India, Indonesia, Nepal, Malaysia, Bangladesh and Myanmar. Some of the countries have been much less studied in this devastating pandemic. This study aims to assess the impact of the Omicron variant in these six countries and estimate the infection fatality rate (IFR) and the reproduction number ${R}_{0}$ in these six South Asia, Southeast Asia and Oceania countries.

**Methods:**

We propose a Susceptible-Vaccinated-Exposed-Infectious-Hospitalized-Death-Recovered model with a time-varying transmission rate $\beta (t)$ to fit the multiple waves of the COVID-19 pandemic and to estimate the IFR and ${R}_{0}(t)$ in the aforementioned six countries. The level of immune evasion and the intrinsic transmissibility advantage of the Omicron variant are also considered in this model.

**Results:**

We fit our model to the reported deaths well. We estimate the IFR (in the range of 0.016 to 0.136%) and the reproduction number ${R}_{0}(t)$ (in the range of 0 to 9) in the six countries. Multiple pandemic waves in each country were observed in our simulation results.

**Conclusions:**

The invasion of the Omicron variant caused the new pandemic waves in the six countries. The higher ${R}_{0}(t)$ suggests the intrinsic transmissibility advantage of the Omicron variant. Our model simulation forecast implies that the Omicron pandemic wave may be mitigated due to the increasing immunized population and vaccine coverage.

**Supplementary Information:**

The online version contains supplementary material available at 10.1186/s12879-023-07984-9.

## Background

The prevalent coronavirus 2019 (COVID-19) pandemic, which was caused by severe acute respiratory syndrome coronavirus 2 (SARS-CoV-2), has been causing substantial infections and deaths since it was first reported in late 2019. RNA viruses mutate quickly and evolve to adapt and survive in changing environments over time. SARS-CoV-2 has muted several times, and there are now five VOCs (Variants of Concern) according to the WHO classification: which are Alpha (Lineage B.1.1.7), Beta (Lineage B.1.351), Gamma (Lineage P.1), Delta (Lineage B.1.617.2), and Omicron (Lineage B.1.1.529).

The Omicron variant was first identified in November 2021 in Botswana and South Africa [1] and soon after was declared as a VOC by the WHO [2]. The most concerning characteristic of the Omicron variant is the constellation of more than 50 mutations [3, 4]. These mutations enhanced its transmissibility and immune evasion ability. Although a study [5] suggests that the Omicron variant may have a smaller chance of causing hospitalization or death than the Delta variant, Omicron (all subvariants) still has a higher reinfection rate than other COVID-19 variants [6].

The study [7] indicated that the COVID-19 pandemic in South Asia (SA) and Southeast Asia (SEA) was mild in 2020 compared with other hot spots, such as Europe or North America. Nevertheless, COVID-19 has caused a health crisis for citizens in SA and SEA countries [8, 9], especially in low- and middle-income countries such as India, Indonesia, Nepal, Malaysia, Bangladesh and Myanmar. These six countries have been experiencing several pandemic waves since late 2019 or early 2020. Furthermore, a sudden increase in the numbers of confirmed cases and deaths caused by the appearance of Omicron totally changed the nature of this pandemic in this region [10].

Seroepidemiology is a valuable tool to investigate the transmission of COVID-19 and reveal undetected infections in populations. The number of confirmed cases cannot accurately reflect the number of actual infections due to the lack of proper diagnoses, variations in testing practices, timing of sampling, or the clinical spectrum of disease. Thus, a seroepidemiological survey and its results could better measure the infection fatality rate and reflect the amplitude of SARS-CoV-2 exposure in the population [11–13].

Many seroepidemiological surveys have been conducted in the above six SA and SEA countries (India, Indonesia, Nepal, Malaysia, Bangladesh and Myanmar) to estimate the severity and assess the trend of the COVID-19 pandemic before the appearance of the Omicron variant in these six countries.

India has conducted four serial population-based serosurveys to estimate the proportion of Indian citizens infected with COVID-19 and to monitor the pandemic trends over time from May 2020 to July 2021. The first two serial population-based serosurveys indicated that the seroprevalences of COVID-19 were 0.73% in May–June 2020 and 7.1% in August–September 2020 [14]. The results of the third serosurvey revealed that 24.1% of India’s population aged > 10 years had been exposed to the SARS-CoV-2 virus by December 2020–January 2021 [15]. The fourth COVID-19 national serosurvey (June–July 2021) [16] found that more than two-thirds of (67.6%) the Indian population developed antibodies against the SARS-CoV-2 virus, and this population developed both natural immunity and vaccine-induced immunity. Despite the fact that the serosurvey results show that a large portion of India’s population has been infected before, Omicron soon became the dominant variant in at least parts of India compared to other variants by late December 2021/early January 2022 [17]. For example, Omicron (BA.2) already made up almost 80% of the infections in Kolkata in late December 2021 [18].

One seroepidemiological study [11], which was conducted in East Java, Indonesia, revealed that the overall prevalence of anti-SARS-CoV-2-IgG (indication of SARS-CoV-2 infection) was 11.4% (207/1,819). The first COVID-19 case in Indonesia was discovered in March 2020. Despite all the social restrictions implemented in Indonesia, the pandemic seems not to be mitigated at all. The increase in the number of confirmed cases and deaths causes Indonesia to have the worst situation among its Southeast Asian counterparts [19, 20]. Omicron’s appearance made the country’s situation much worse.

A longitudinal seroprevalence study [21] based on hospital health workers in the Kathmandu valley, Nepal, found that the overall posterior predictive hospital-wise seroprevalence ranged between 38.1% (95% CrI 30.7.0–44.1%) and 40.5% (95% CrI 34.7–47.0%). This result suggested that a substantial proportion of health workers in Kathmandu had been infected with COVID-19 by the end of 2020. The first COVID-19 case was detected on 23 January 2020, and COVID-19 soon spread all over Nepal despite the strict interventions implemented by the government [22]. The new COVID-19 wave driven by the Omicron variant started in January 2022, and some experts believe that this new wave of infections was triggered by the increase in the number of confirmed cases in neighboring India [23–26]. A new record of over 10,200 new daily infections was reported on January 18, 2022. This number means that there were 900 daily new cases more than the previous high in May 2021 when the Delta variant dominated the wave.

A series of serosurveys have been conducted in Malaysia [27–30]. One study [27] estimated that the seroprevalence among health workers was 4.5% in March 2020. The study [28] indicated a 0.4% seroprevalence from January to June 2020 based on the serum samples collected at a teaching hospital serving Kuala Lumpur and Selangor state. The study [29] concluded a 0.0% seroprevalence among health workers from April to May 2020. Another serosurvey [30] conducted from July to September 2021 in Malaysia suggested that the seroprevalence was 99.9% among migrant workers but was only 12.1% among local workers. The results had differences in ranges. The low seroprevalence of the first three surveys may be attributed to the early timing (all three were conducted in 2020, which was the early beginning of COVID-19 in Malaysia). Malaysia has experienced four COVID-19 pandemic waves from the beginning of the pandemic in February 2020 until October 2021. The first COVID-19 Omicron case was reported on 2 December 2021 in Malaysia [31]. The breach of the quarantine order accelerated the spread of Omicron, and the new pandemic wave was on its way.

One seroprevalence study [32], which was conducted in the Chattogram Metropolitan Area, Bangladesh, from February to September 2021 and studied health workers, in and outpatients, and garment workers, estimated the overall seroprevalence to be 66.99% (95% CI: 63.40–70.40%). Another longitudinal cohort study [33] compared the serological response in patients who had differing severities of COVID-19 infection. The COVID‐19 pandemic situation in Bangladesh was under control, and the government intended to lift the restrictions on public gathering and movement in August 2021 [34]. However, the first report of an Omicron case on December 12, 2021, in Bangladesh forced the government to issue more restrictions to contain this new surge of infections [35].

Even though there aren’t serosurveys that have been conducted in Myanmar currently, the other data could also enable us to estimate the severity of the COVID-19 pandemic in Myanmar. One study [8] concluded that Myanmar recorded the highest CFR (3.8%) as of 17 September 2021 and had the lowest COVID-19 vaccine coverage as of 28 December 2021 in the region (within 11 countries of Southeast Asia). Myanmar experienced one of the most severe COVID-19 outbreaks in Southeast Asia by late 2020. The collapse of the COVID-19 testing system and vaccination deployment in February 2021 worsened the situation [36].

The above serosurveys revealed the severity of the COVID-19 pandemic in these six countries that substantial number of individuals have been exposed to or infected by the SARS-Cov-2. Further, we intend to adopt the mathematical models to investigate the COVID-19 transmission dynamics and provide more detailed estimation of the COVID-19 pandemic severity in these countries. Many works on investigating and forecasting the transmission dynamics of COVID-19 pandemic by mathematical modeling methods have been published due to the prevalence of COVID-19 and the surge of the Omicron variant.

Wang et al. [37] proposed an SVEIHR model to investigate the relationships between different model parameter settings and hospitalized cases in Hong Kong Special Administrative Region, China. And herd immunity threshold for the ancestral, Delta, and Omicron strains. Oh et al. [38] derived SEIQRDVP and SEIQRDV3P models to estimate the fatality, morbidity, and transmission rates and affect vaccine efficacy of the Omicron variant in South Korea. Only one vaccine level was considered in the SEIQEDVP model while three vaccination levels were considered in the SEIQRDV3P model. They concluded that the SEIQRDV3P model showed better simulation results and they suggested that the rapid rise in COVID-19 cases would continue. An ecological study [39] was conducted to explore the country-level morbidity of Omicron infection and the spatial transmission of the Omicron variant. Both spatial analysis and temporal analysis were conducted in the study. Negative correlations and positive correlations between the morbidity of COVID-19 and others factors were observed in this study. They also found spatial clustering patterns of the Omicron variant infection.

Many nonpharmaceutical interventions (NPIs) were implemented to reduce the rapid spread of COVID-19. But the effectiveness of these NPIs remains unclear. Mathematical modeling and optimal control approach were adopted to access the effects of three different time-independent interventions on COVID-19 transmission dynamics [40]. They concluded that the interventions or the measures against COVID-19 could highly reduce infection cases if these interventions were implemented optimally. A study [41] was conducted to investigate the influence of passenger air traffic on COVID-19 transmission. They applied many models such as Poisson model, quasi-Poisson model, Negative binomial model, zero-inflated models, and Hurdle models to model counting variables and implement cross-validation. They conclude that passenger air traffic may facilitate COVID-19 transmission. They also suggested that counting variables models can be applied to study COVID-19 transmission.

Besides the classical SIR-based mathematical models, the machine learning method was also applied to explore the transmission dynamics of the COVID-19 pandemic. Machine learning technic was applied in a study [42] to find the best models for the COVID-19 cumulative cases forecasting. They focused on the three univariate models: ARIMA, ESM and ETS. And they concluded that the most appropriate for the COVID-19 cumulative cases forecasting was ETS because it had the smallest bias for the forecasting.

Mathematical models have been widely adopted to study the COVID-19 transmission dynamics since the very beginning of this pandemic. The above seroprevalence studies conducted in these six countries revealed the harsh reality that the COVID-19 pandemic was severe. Although these countries have been experiencing multiple COVID-19 pandemic waves before, the appearance of the Omicron variant would make these six countries suffer more. This study estimates the impact of the Omicron variant in six countries, India, Indonesia, Nepal, Malaysia, Bangladesh and Myanmar, by applying a mathematical model based on the original SIR model.

## Methods

Mathematical models have been successfully adopted to study the mechanisms and to predict the transmission dynamics of infectious diseases. The mathematical modeling of COVID-19 is crucial to mitigate pandemic transmission by suggesting possible and optimal interventions. The basic assumptions along with the collected data could enable us to use the mathematical model to forecast the current COVID-19 pandemic trends and facilitate the public health agency's policymaking.

We propose an S-${S}_{V}$-E-I-H-D-R model to simulate the multiple mortality waves of COVID-19 in six countries in South Asia, Southeast Asia and Oceania. The level of immune evasion and the intrinsic transmissibility advantage of the Omicron variant are also estimated in this model.

**Fig. 1:**
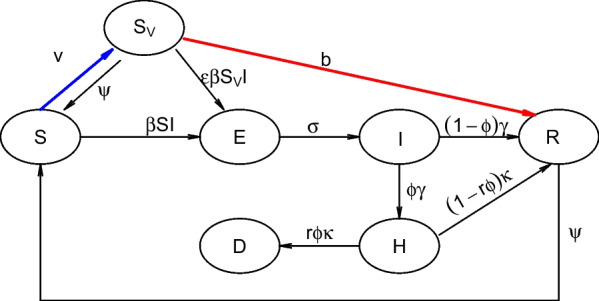
Flow chart of the S-${S}_{V}$-E-I-H-D-R model

The population was divided into susceptible (S), vaccinated (${S}_{V}$), exposed (E), infectious (I), hospitalized/delayed (H), recovered/immunized (R), and death (D) classes. The waning of infection-induced and vaccine-induced immunity, both the second dose and the third (booster) dose, and the immune evasion of Omicron are all considered in our model. As shown in Fig. 1, the susceptible class would be moved to the exposed class after exposure to the SARS-COV-2 virus, or some of the susceptible individuals would be vaccinated and would be moved to the vaccinated class. Some of the vaccinated class may lose their immunity and return to the susceptible class or may directly be moved to the recovered/immunized class. It is also possible for some of the vaccinated individuals to be moved to the exposed class after being exposed to the virus. The exposed class would be moved to the infectious class after a latency period. Some of the infectious class would either be moved to the hospitalized/delayed class or could be directly moved to the recovered/immunized class. The individuals in the hospitalized/delayed class may die and be moved to the death class or recover and be moved to the recovered/immunized class. Some of the hospitalized/delayed individuals may lose their immunity to be susceptible again.

1 $$\begin{array}{l}\frac{dS}{dt}=-\frac{\beta SI}{N}-vS+\psi {S}_{V}+\psi R,\\ \frac{d{S}_{V}}{dt}=-\frac{\epsilon \beta {S}_{V}I}{N}+vS-\psi {S}_{V}-b{S}_{V},\\ \frac{dE}{dt}=\frac{\beta \left(S+{S}_{V}\right)I}{N}-\sigma E,\\ \frac{dI}{dt}=\sigma E-\gamma I,\\ \frac{dH}{dt}=\phi \gamma I-\kappa H,\\ \frac{dD}{dt}=r\phi \kappa H,\\ \frac{dR}{dt}=\left(1-\phi \right)\gamma I+\left(1-r\phi \right)\kappa H+b{S}_{V}-\psi R.\end{array}$$

As mentioned above, we denote the susceptible, vaccinated, exposed, infectious, hospitalized/delayed, recovered/immunized, and death classes as S, ${S}_{V}$, E, I, H, R, and D in our model, respectively. Parameter $N$ denotes the population of each country. We denote ${t}_{0}$, ${t}_{1}$, and ${t}_{2}$ as the start time of the study period, time of immune evasion, and end time of the study period, respectively. The transmission rate $\beta \left(t\right)$ is a time-varying function. Based on previous studies [10, 43–49], $\beta \left(t\right)$ is assumed to be an exponential cubic spline (i.e., $\beta \left(t\right)$=exp(cubic_spline)) with 16 nodes (see Additional file 1: Table S3 or 17 nodes see Additional file 1: Figs. S1–S3). Twelve nodes fall between $\left[{t}_{0},{t}_{1}\right]$, and four nodes are evenly distributed over $\left[{t}_{1},{t}_{2}\right]$. A jump in the transmission rate $\beta (t)$ at ${t}_{1}$ is allowed, and the level of the jump is estimated. Parameter $v$ is the vaccination rate (second dose), and $b$ is the booster rate (third dose). The vaccination data, which we obtained are in the form of per capita (the proportion of vaccinated individuals among the whole population), cannot be directly incorporated into our model. What we can incorporate into our model is the proportion of vaccinated individuals among the susceptible population. Thus, we translated the vaccination data from per capita to per unvaccinated (the proportion of the vaccinated among the unvaccinated), The booster data are translated from per capita to the per fully vaccinated (i.e., second dose receiver) for the same reason. It is worth mentioning that a seven-day delay is included for the second dose, and no delay is included for the booster. We consider this delay when we input vaccination data (as a covariate) into the model. These results are due to the observed delay of vaccine protection turn-on time [50]. We set the relative susceptibility of vaccinated vs. unvaccinated $\epsilon$ as 0.1 and the rate of loss of immunity protection $\psi$ as 0.5 or 0.333. The parameters $\sigma ,\gamma ,\kappa$ denote the rate of infectiousness onset after exposure, rate of loss of infectiousness, and rate of recovery from the severity stage, respectively. We set $\sigma =365/2 per year$, $\gamma =365/3 per year$, $\kappa =365/12 per year$ [44, 51–53]. That is, we have a 2-day latent period, a 3-day infectious period and a 12-day delay from the loss of infectiousness to death. The pre-symptomatic occurs in this interval (from 2 to 5 days) Since we used a short latent period (2 days) and the incubation period is 5 days. And, we included both asymptomatic and symptomatic cases in the infections class [54]. The infection severity case ratio and severity case mortality ratio, which have the same values in our model, are set as $\phi$ (see Additional file 1: Table S2). $r$ is a scaling factor, we set $r=1$ before time ${t}_{1}$, and $r$ would linearly decrease from 1 to $\alpha$ (for detailed information about $\alpha$ see Additional file 1: Table S2) during a period of time $[{t}_{1},{t}_{1}+36 days]$.

The parameter $\phi$ denotes the ratio of severe cases out of all infected cases. Due to the unavailability of COVID-19 hospitalized severe cases data, the proportion of mortality out of severe cases also was defined as $\phi$. Hence the overall IFR is equivalent to the ${\phi }^{2}$. The possible reason for making this assumption is that the exact definitions of H and $\phi$ are not important, since we only fit the death data rather than the hospitalized cases or infected cases. Actually, the compartment H would serve as an intermediate class between the infected class and the death class.

We quantify the transmission rate using $\beta (t)/\gamma$, and we call it the time-varying reproductive number and we denote it as ${R}_{0}(t)$. Note that this is the basic reproductive number at the beginning of the pandemic, and the immunity unadjusted reproductive number in the middle of the pandemic. The effective reproductive number is $\beta (t)S(t)/\gamma$*.* We set the initial ${R}_{0}<3$ and ${R}_{0}(t)<8$ at each cubic spine node. ${R}_{0}(t)$ would increase at time ${t}_{1}$ and is assumed to be $>4$ at and after time ${t}_{1}$.

Moreover, we defined the weekly death ${D}_{t+\Delta t}$ as ${\int }_{t}^{t+\Delta t}r\kappa Hdt$. The weekly reported deaths are defined as ${Z}_{t+\Delta t}$ and we assume

$${Z}_{t+\Delta t}\sim NegativeBinomial \left(mean={D}_{t+\Delta t}, variance={D}_{t+\Delta t}\left({1+\tau D}_{t+\Delta t}\right)\right),$$

Then we defined the log-likelihood function as:

$$Log\_Likelihood={\sum }_{i=1}^{n}log f\left({Z}_{i}|{Z}_{1:i-1},\Theta \right).$$

The population is assumed to be constant during our study period. The timescale of current COVID-19 is much shorter than the demographic time scale [48, 55, 56]. Thus, the demographic processes (i.e., births and natural deaths processes) are not included in this model. The log-likelihood values, log-likelihood values' standard derivations, and the specific numbers of populations are shown in Additional file 1: Table S1.

We assume that the pre-Omicron IFR is between 0.011% and 0.25%. The Omicron IFR is assumed to be reduced by a maximum of 90%. Thus, we estimated the reduction in IFR of Omicron relative to the IFR of pre-Omicron with a max of 90% (namely < 90% reduction). We assume a linear change in IFR from pre-Omicron to the Omicron IFR from ${t}_{1}$ (the time of Omicron intro) to ${t}_{1}+36 days$. The pre-Omicron IFR is shown in $\left[{t}_{0},{t}_{1}\right]$. The Omicron IFR is from ${t}_{1}+36 days$ to ${t}_{2}$ and between ${t}_{1}$ and ${t}_{1}+36 days$ is a linear transition from the pre-Omicron IFR to the Omicron IFR. We assume a sudden loss of immunity at time ${t}_{1}$ due to the immune evasion of the Omicron variant and a breakpoint of the transmission rate at time ${t}_{1}$.

We first considered a ‘single’ invasion scenario of Omicron variants in the model simulation. Then we considered multiple (e.g., ‘twice’) invasion scenario by the initial Omicron BA.1 (or BA.2 or both) variant and the follow-up Omicron (BA.4 or BA.5) variant, to compare with the single invasion scenario. We set $\theta$ as the first immune evasion proportion and ${\theta }_{1}$ as the second immune evasion proportion. $\theta$ and ${\theta }_{1}$ represent the loss of immunity for the recovered (R) and vaccinated (V) at time ${t}_{1}$ and ${t}_{1}+120 days$, respectively. We presumed that the first Omicron occurred at time ${t}_{1}$ and the second invasion occurred at ${t}_{1}+120 days$ in each country for simplicity. $\alpha$ is the relative ratio of the Omicron IFR vs. the pre-Omicron IFR (For the specific parameter values related to either a ‘single’ invasion or two invasions, see Table 1 and Additional file 1: Table S2).

**Table 1:** Parameters utilized in the model

Parameter		Unit	
$N$	Population of each country		
$\beta$	Time-varying transmission rate	Per year	$\beta =\gamma {\mathcal{R}}_{0}$
$v$	Vaccination rate (second dose)	Per day	Translate from data (per capita) to per unvaccinated
$\psi$	Rate of loss of immunity protection	Per year	0.333 or 0.5
$\varepsilon$	Relative susceptibility of vaccinated vs. unvaccinated		0.1
$b$	Booster rate (third dose)	Per day	Translate from data (per capita) to per second dose susceptible
$\sigma$	Rate of infectiousness onset after exposure	Per year	365/2
$\gamma$	Rate of loss of infectiousness	Per year	365/3
$\phi$	Infection Severity Case ratio and severity case mortality ratio		[0.04,0.09]
$\kappa$	Rate of removal from severity stage	Per year	365/12
$r$	Severity Case mortality ratio		$r=1$ before time ${t}_{1}$, and $r$ would linearly decrease from 1 to $\alpha$ during a period of time $[{t}_{1},{t}_{1}+36 days]$
${t}_{0},{t}_{1}, {t}_{2}$	Start time of study period, Time of immune evasion, end time of study period		${t}_{0}$ is February 11, 2020, ${t}_{1}$ to be estimated and is around Jan 1, 2022, ${t}_{2}$ is June 6, 2022

All data we deploy in our model simulation, which include the reported COVID-19 death data and vaccination data (second dose and third dose/booster), are from the website ‘Our World in Data’ [57–60]. We fit our model to the 122 weeks of data from February 11, 2020, to June 6, 2022. We choose the data from June 7, 2022, to August 29, 2022, as the testing part to test our model prediction. In this testing period, we assume that the second dose coverage will increase by an additional 10% and the booster coverage will increase by an additional 20%. R statistical language and the well-known R package POMP were used to analyze and process the data. To be specific, we adopted a partially observed Markov process (POMP) model applying a maximum likelihood-based iterated filtering technique to fit the mortality data. Detailed information on POMP and how to apply it to epidemiological models can be found in previous studies [61–63]. Moreover, I want to make it clear that even though our base model has a deterministic model, when simulating this work to mimic the reality, we include process noise (via Euler multinomial algorithm) and observational noise (negative binominal distribution). Thus, our full model is a stochastic model [64].

## Results

**Fig. 2:**
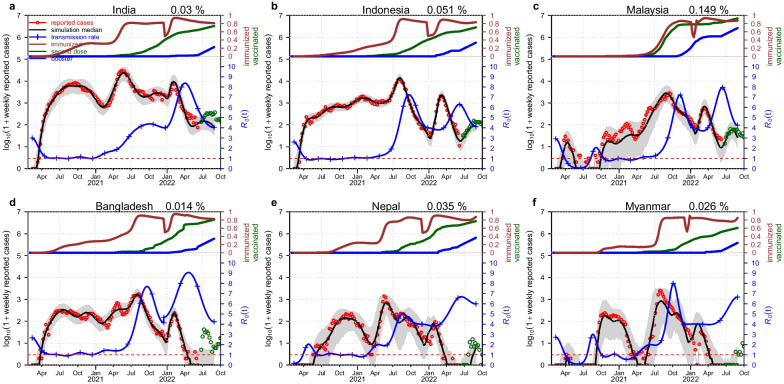
Model simulated death vs. reported death with the assumption of only one Omicron invasion and the rate of loss of immunity protection $\psi =1/2$ in India (**a**), Indonesia (**b**), Malaysia (**c**), Bangladesh (**d**), Nepal (**e**) and Myanmar (**f**). The brown curve, green curve and blue curve at the top of each panel show the currently immunized people per capita, the number of persons who received a second dose per capita and the number of persons who received a booster dose per capita, respectively. The sudden drop in the brown curve shows the immune evasion caused by one Omicron variant invasion. The red circles, black curve and blue cure with the plus sign at the bottom of each panel show the reported deaths, the simulation median and the transmission rate (in the unit of ${R}_{0}(t)=\beta (t)/\gamma$), respectively. The grey region denotes the 95% CI of 1000 model simulations. The green circles that overlap the black curve are the reported deaths for testing part. The extra green circles are the reported deaths to show the COVID-19 pandemic trends. The percent on the top of each panel is the estimated maximum log-likelihood pre-Omicron IFR

In Fig. 2, the brown curve denotes the ratio of the currently immunized population among the whole population. We can see that the brown curve had an abrupt drop in January 2020. This was exactly the time that the Omicron variant was prevalent in these countries. Therefore, this abrupt drop shows the immune evasion of the Omicron variant, i.e., a proportion of the immunized people lost protection (moving to S) due to the immune evasion ability of the Omicron variant. The green curve (second dose per capita) and the blue curve (booster per capita) always increased, which indicates that all six countries deployed COVID-19 vaccines as a main pharmaceutical intervention to prevent citizens from being infected by COVID-19. The blue curve with the plus sign clearly shows the transmission ability of the Omicron variant. A sudden increase in the transmission rate (in the unit of ${R}_{0}(t)=\beta (t)/\gamma$), which indicates an advantage in the intrinsic transmissibility of Omicron, can be observed in January 2022. The transmission rate peaked around April 2022 and then started to decrease. The fluctuation of the transmission rate in these countries is not simultaneous because the Omicron variant hit and became the dominant variant in different countries during different periods. However, this fluctuation of the transmission rate coincides with the Omicron variant surge time in each country. The red circles denote the reported deaths. We can see that the numbers of reported deaths were always high when the Omicron variant dominated the COVID-19 pandemic wave. Furthermore, we can see that the change in the transmission rate can reflect the weekly reported deaths. The black curve is the model simulation median. From Fig. 2, we can see that our model fitting is reasonably good; it can fit the multiple COVID-19 pandemic waves in each country.

The green circles that overlap the black curve are the reported deaths for testing part. We choose the data from June 7, 2022, to August 29, 2022, as the testing part to test our model prediction. From Fig. 2, we can see that the test is acceptable in some countries, as the green circles are in the grey region. In the other countries, the model prediction is not as satisfactory, as the green circles are out of the grey region. This unsatisfactory model prediction may be attributed to the immune evasion of the new Omicron sublineages. We only considered the emergence of Omicron BA.1, and the subsequent surge of BA.2, BA.2.12, BA.4 and BA.5 is omitted in this simulation. The short-term forecast was difficult, as we showed here. The recent increase in deaths and confirmed cases could be due to the invasion by a new Omicron variant (BA.4 or BA.5). Knowledge of the magnitude of the immune evasion of BA.4 or BA.5 is crucial for a correct forecast. Moreover, the extra green circles are for readers to see the COVID-19 pandemic trends.

The percent on the top of each panel is the estimated maximum log-likelihood pre-Omicron IFR. Peculiarly, the IFR estimated in Malaysia is higher than those in the other five countries. The economic level and public health condition are better in Malaysia than in the other five countries.

**Fig. 3:**
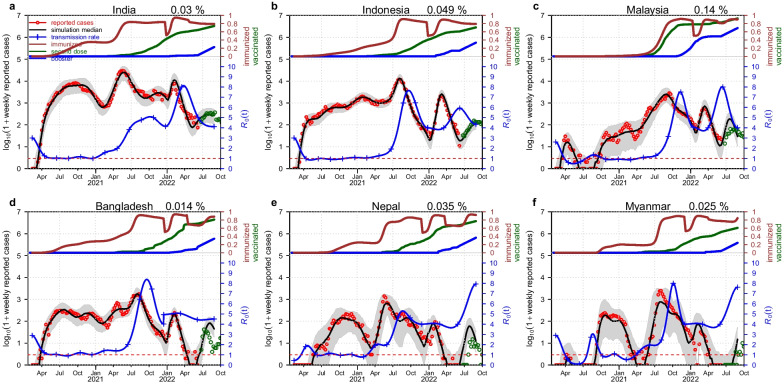
Model simulated death vs. reported death with the assumption of two Omicron invasions and the rate of loss of immunity protection $\psi =1/2$ in India (**a**), Indonesia (**b**), Malaysia (**c**), Bangladesh (**d**), Nepal (**e**) and Myanmar (**f**). The brown curve, green curve and blue curve at the top of each panel show the currently immunized people per capita, the number of persons who received a second dose per capita and the number of persons who received a booster dose per capita, respectively. The sudden drops in the brown curve demonstrate the immune evasion caused by two Omicron variant invasions. The red circles, black curve and blue curve with the plus sign at the bottom of each panel show the reported deaths, the simulation median and the transmission rate (in the unit of ${R}_{0}(t)=\beta (t)/\gamma$), respectively. The grey region denotes the 95% CI of 1000 model simulations. The green circles that overlap the black curve are the reported deaths for testing part. The extra green circles are the reported deaths to show the COVID-19 pandemic trends. The percent on the top of each panel is the estimated maximum log-likelihood pre-Omicron IFR

Figure 3 shows another scenario with the assumption of two Omicron invasions. The basic simulation results are similar to Fig. 2, which means that our model simulation well matched the data in each scenario. The main difference from Fig. 2 is that we consider not only the invasion of Omicron BA.1 and BA.2 but also the following emergence of BA.4 and BA.5 in this simulation. From Fig. 3, we can see that these simulation results are much better than those in Fig. 2, especially for the reported deaths. Furthermore, we considered different scenarios to fit our model, such as one Omicron invasion or two Omicron invasions, and a cubic spline with 17 nodes or 16 nodes, and set the different values of $\psi$ ($1/2 or 1/3$). The simulation results are akin to Figs. 2 and 3 (see Additional file 1: Figs. S1–S4). There would be some variations in each scenario's simulation results, but the overall results are still consistent. The results imply that two Omicron invasions scenario could be a better explanation for the current COVID-19 pandemic waves in these countries, and our model are not sensitively dependent on these assumptions.

**Fig. 4:**
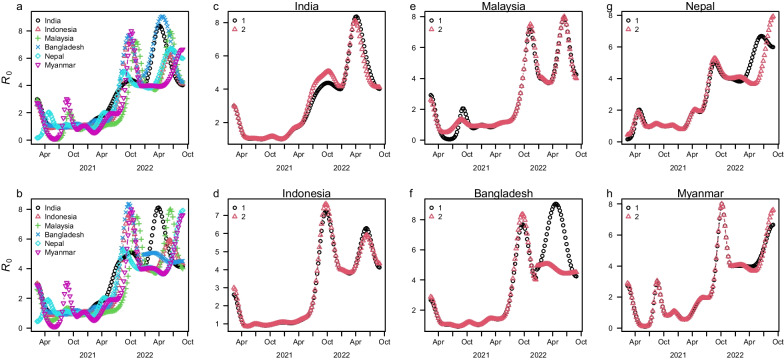
The transmission rate (in the unit of ${R}_{0}(t)$ under the scenarios of a ‘single’ invasion (**a**) vs ‘twice’ invasions (**b**) of the Omicron variants. The comparison of transmission rate between a ‘single’ invasion scenario and ‘twice’ invasions scenario of the Omicron variants in India (**c**), Indonesia (**d**), Malaysia (**e**), Bangladesh(**f**), Nepal (**g**), and Myanmar (**h**)

In Fig. 4, we compare the changes in the transmission rate in the different scenarios: the ‘single’ invasion scenario versus the ‘two’ invasions scenario of Omicron variants. There are changes in ${R}_{0}(t)$ before the time of the second Omicron invasion, but they are broadly consistent under these two scenarios. However, ${R}_{0}(t)$ significantly varied in many countries at the time of the second Omicron invasion. ${R}_{0}(t)$ was higher after the Delta invasion than before the Delta invasion. From (a) and (b) in Fig. 4, we can see that ${R}_{0}(t)$ increased early in India, followed closely by increases in Nepal and Bangladesh. This is due to the early invasion of the Delta variant. The Delta variant was first detected in India in late 2020-early 2021 and soon spread to neighboring and other countries. Then ${R}_{0}(t)$ peaked during the first Omicron invasion period. Under the ‘single’ invasion scenario, we can observe that there is only one peak of ${R}_{0}(t)$ in panel a of Fig. 4. In contrast, we can observe that there are two peaks of ${R}_{0}(t)$ in panel b of Fig. 4. The appearance of another peak of ${R}_{0}(t)$ clearly demonstrates the significant immune evasion ability and transmissibility of almost all Omicron sublineages.

## Discussion and conclusion

We proposed an SEIR-based model with a time-varying transmission rate to simulate and forecast the COVID-19 pandemic in India, Indonesia, Nepal, Malaysia, Bangladesh and Myanmar. Some of these countries have been much less studied in this pandemic (the specific explanation of the less studied countries see Additional file 1: Table S5). This research could provide a comprehensive understanding of the COVID-19 pandemic in less-studied countries. This model can be simply applied to any other country.

We estimated the basic reproduction number and the IFR of the COVID-19 pandemic in these six countries. We found that the basic reproductive number ${R}_{0}(t)$ increased to a high level (as high as 9–10) in India, Bangladesh and Nepal by August-October 2021. ${R}_{0}(t)$ was below 5 in Indonesia, Malaysia and Myanmar in 2021, and reached a high value (as high as 8–10) later. These results show that the transmission of the Delta variant (July–December 2021) and the Omicron variant (after Dec 2021) was intensive. Moreover, the comparison between ‘one invasion scenario’ and ‘two invasions scenario’ shows that the ‘two invasions scenario’ could better explain the current pandemics waves under the same conditions.

The immune evasion of the Omicron variant increases the size of the susceptible pool, and together with the increase in the intrinsic transmission rate, the immune evasion causes the wave of Omicron. Based on fitting the model to the data, these two effects would be difficult to disentangle. Here, we find that immune evasion is more evident (a trough in the brown curve of Figs. 2 and 3), than an increase in the intrinsic transmission rate (a jump in the blue curve). In most countries a trough in the brown curve (estimated) is evident. Pulliam et al. [65] found that Omicron had evident immune evasion, but there was no population-level immune evasion of the pre-Omicron variants. This finding justified our model choice. We allow for the natural loss of immunity but not for the sudden loss of immunity for pre-Omicron variants. However, one study [66] estimated that the immune escape from the prior wild-type infection was $34.6\%$ ($95\%$ CI: 0–64.2%), which contradicts the study [65]. We think Pulliam et al. [65] is more sound and in line with many other studies [67, 68]. Moreover, the model simulation result of the transmission rate contains a short forecast period and shows a decreasing trend in these countries. This decreasing trend repeats the information of the previous Delta variant and suggests that the pandemic wave dominated by the Omicron variant will eventually be mitigated.

We set the decay of immunity (both due to infection or vaccination) as 0.5 per year. We assume that the observed infection fatality ratio (IFR, which involves the underreporting of infections, but assumes that deaths are reliable) ranges from 0.0156 to 0.249%. We ignored the possible reduced IFR during the Omicron wave. We estimate that the pre-Omicron IFRs in these six countries are 0.03% (India), 0.049% (Indonesia), 0.14% (Malaysia), 0.014% (Bangladesh), 0.035% (Nepal) and 0.028% (Myanmar) (IFR in Fig. 3). Among them, Malaysia has the highest IFR of 0.141%, which may suggest that the underreporting of deaths in Malaysia could be the lowest among these countries. Malaysia is more economically developed and has a better public health condition than the other five countries. In a previous study [49], the authors indicated that the IFR in India was heavily underestimated. Jha et al. [69] found that India’s cumulative COVID deaths by September 2021 were six to seven times higher than officially reported. The comparison between Indonesia and Malaysia is also striking, although they are close both in religion and geography. This may also be ascribed to the difference in GDP or public health condition. We set the IFR of the Omicron variant as half of the pre-Omicron IFR. The IFR we estimated assumes that the death data are absolutely correct. Even though we reference some serological studies, the constraint of the sample size and the sampling method may cause deviation in the final results. We found that the underestimation of IFR is a common phenomenon in this region, not just in India. This low IFR may be attributed to the underreporting along with some other unknown factors. One study [70] could be valid evidence to support our conclusion. The higher ratio between the excess mortality rate and reported COVID-19 mortality rate (see Additional file 1: Table S4) may represent a higher level of underreporting of death data. The ratio of excess death vs. reported death is 1.58 (the lowest among these six countries) in Malaysia, which indicates the lowest underreporting of death data in Malaysia. The high ratio in other countries suggests severe underreporting. Moreover, the IFR would be more reasonable when we multiply our estimated IFR by this ratio.

The one strength of this work includes that we fitted a simple model to the reported death with a flexible time-varying transmission rate, which was assumed to be an exponential cubic spline function to reflect the COVID-19 transmission ability and the impact of COVID-19 interventions simultaneously. We also tried the different scenarios of Omicron invasions and we found that the two invasions could better approach the reality. Besides the strengths, this study also has several limitations. One most obvious limitation is that all parameters were assumed to be constant except for the transmission rate. This model only focused on the whole country while the heterogeneity across regions was neglected. And, we only relied on the reported data and adopted a non-mechanistic cubic spline function for the transmission rate. For further study, incorporating all kinds of control measures (e.g., google mobility matrix, etc.) could be considered.

In conclusion, we propose a simple SEIR-based model to simulate and fit the COVID-19 pandemic trends in six South Asian, Southeast Asian and Oceanic countries. This work is a multiple-country study, and some of the countries, such as Malaysia, Bangladesh and Myanmar, are less studied in previous research studies. We considered the multiple invasions of Omicron variants that elicited immune evasion effects in these countries’ population, and we found the ‘two invasions scenario’ could better explain the current pandemic waves. The simulation results are sound. Moreover, this is probably the simplest model that has examined and simulated the waves in these countries.

## Supplementary Information

**Additional file 1: Table S1.** The population, Log likelihood value and Log likelihood value standard derivation. **Table S2.** The parameters in the model simulation. **Table S3.** The cubic spline nodes number and nodes value of the transmission rate β(t) (log.β_i_, i = 1, . . . , 16). **Table S4.** The ratio of excess death vs report deaths and its 95% CI. **Table S5.** Literature statistics under different keywords and search settings. **Figure S1.** Model simulated death vs. reported death with the assumption of only one Omicron invasion and the rate of loss of immunity protection ψ = 1/2 in India(a), Indonesia(b), Malaysia(c), Bangladesh(d), Nepal(e) and Myanmar(f). The brown curve, green curve and blue curve at the top of each panel show the currently immunized people per capita, the number of persons who received a second dose per capita and the number of persons who received a booster dose per capita, respectively. The sudden drop in the brown curve shows the immune evasion caused by one Omicron variant invasion. The red circles, black curve and blue cure with the plus sign at the bottom of each panel show the reported cases, the simulation median and the transmission rate (in the unit of R0(t) = β(t)/γ), respectively. The grey region denotes the 95% CI of 1000 model simulations. The green circles that overlap the black curve are the reported deaths for testing part. The extra green circles are the reported deaths to show the COVID-19 pandemic trends. The percent on the top of each panel is the estimated maximum log likelihood pre-Omicron IFR. **Figure S2.** Model simulated death vs. reported death with the assumption of only one Omicron invasion and the rate of loss of immunity protection ψ = 1/3 in India(a), Indonesia(b), Malaysia(c), Bangladesh(d), Nepal(e) and Myanmar(f). The brown curve, green curve and blue curve at the top of each panel show the currently immunized people per capita, the number of persons who received a second dose per capita and the number of persons who received a booster dose per capita, respectively. The sudden drop in the brown curve shows the immune evasion caused by one Omicron variant invasion. The red circles, black curve and blue cure with the plus sign at the bottom of each panel show the reported cases, the simulation median and the transmission rate (in the unit of R0(t) = β(t)/γ), respectively. The grey region denotes the 95% CI of 1000 model simulations. The green circles that overlap the black curve are the reported deaths for testing part. The extra green circles are the reported deaths to show the COVID-19 pandemic trends. The percent on the top of each panel is the estimated maximum log likelihood pre-Omicron IFR. **Figure S3.** Model simulated death vs. reported death with the assumption of two Omicron invasions and the rate of loss of immunity protection ψ = 1/2 in India(a), Indonesia(b), Malaysia(c), Bangladesh(d), Nepal(e) and Myanmar(f). The brown curve, green curve and blue curve at the top of each panel show the currently immunized people per capita, the number of persons who received a second dose per capita and the number of persons who received a booster dose per capita, respectively. The sudden drops in the brown curve demonstrate the immune evasion caused by two Omicron variant invasions. The red circles, black curve and blue curve with the plus sign at the bottom of each panel show the reported cases, the simulation median and the transmission rate (in the unit of R0(t) = β(t)/γ), respectively. The grey region denotes the 95% CI of 1000 model simulations. The green circles that overlap the black curve are the reported deaths for testing part. The extra green circles are the reported deaths to show the COVID-19 pandemic trends. The percent on the top of each panel is the estimated maximum log likelihood pre-Omicron IFR. **Figure S4.** Model simulated death vs. reported death with the assumption of twice Omicron invasions and the rate of loss of immunity protection ψ = 1/3 in India(a), Indonesia(b), Malaysia(c), Bangladesh(d), Nepal(e) and Myanmar(f). The brown curve, green curve and blue curve at the top of each panel show the currently immunized people per capita, the number of persons who received a second dose per capita and the number of persons who received a booster dose per capita, respectively. The sudden drops in the brown curve demonstrate the immune evasion caused by two Omicron variant invasions. The red circles, black curve and blue curve with the plus sign at the bottom of each panel show the reported cases, the simulation median and the transmission rate (in the unit of R_0_(t) = β(t)/γ), respectively. The grey region denotes the 95% CI of 1000 model simulations. The green circles that overlap the black curve are the reported deaths for testing part. The extra green circles are the reported deaths to show the COVID-19 pandemic trends. The percent on the top of each panel is the estimated maximum log likelihood pre-Omicron IFR.

## Data Availability

All the data used can be found in the public domain, available from [71] and https://covid19.who.int/. The scripts and data used to perform the analysis and generate the figures in this paper are available on Zotero: (https://www.zotero.org/groups/4883840/chen_boqiang/library).
