# Supplementary material for: Twice evasions of Omicron variants explain the temporal patterns in six Asian and Oceanic countries
Source: BMC Infect Dis. 2023 Jan 13;23:25. doi: 10.1186/s12879-023-07984-9 (PMC9839219; doi:10.1186/s12879-023-07984-9)
Supplement: Supplementary file 1 — Additional file 1: Table S1. The population, Log likelihood value and Log likelihood value standard derivation. Table S2. The parameters in the model simulation. Table S3. The cubic spline nodes number and nodes value of the transmission rate β(t) (log.βi, i = 1, . . . , 16). Table S4. The ratio of excess death vs report deaths and its 95% CI. Table S5. Literature statistics under different keywords and search settings. Figure S1. Model simulated death vs. reported death with the assumption of only one Omicron invasion and the rate of loss of immunity protection ψ = 1/2 in India(a), Indonesia(b), Malaysia(c), Bangladesh(d), Nepal(e) and Myanmar(f). The brown curve, green curve and blue curve at the top of each panel show the currently immunized people per capita, the number of persons who received a second dose per capita and the number of persons who received a booster dose per capita, respectively. The sudden drop in the brown curve shows the immune evasion caused by one Omicron variant invasion. The red circles, black curve and blue cure with the plus sign at the bottom of each panel show the reported cases, the simulation median and the transmission rate (in the unit of R0(t) = β(t)/γ), respectively. The grey region denotes the 95% CI of 1000 model simulations. The green circles that overlap the black curve are the reported deaths for testing part. The extra green circles are the reported deaths to show the COVID-19 pandemic trends. The percent on the top of each panel is the estimated maximum log likelihood pre-Omicron IFR. Figure S2. Model simulated death vs. reported death with the assumption of only one Omicron invasion and the rate of loss of immunity protection ψ = 1/3 in India(a), Indonesia(b), Malaysia(c), Bangladesh(d), Nepal(e) and Myanmar(f). The brown curve, green curve and blue curve at the top of each panel show the currently immunized people per capita, the number of persons who received a second dose per capita and the number of persons who recei [file 12879_2023_7984_MOESM1_ESM.pdf]

Additional material of Twice evasions of Omicron variants explain the temporal patterns in six Asian and Oceanic countries

Boqiang Chen[1], Yanji Zhao[1], Zhen Jin[2], Daihai He *[1], and Huaichen Li[3]

[1]Department of Applied Mathematics, Kong Kong Polytenic University, Hong Kong, China
[2]School of Computer and Information Technology, Shanxi University, Taiyuan, China
[3]Department of Respiratory and Critical Care Medicine, Shandong Provincial Hospital Affiliated to Shandong First Medical University, Jinan, China

S1 Parameters used in the model simulation

We have introduced some parameters of S-S_V-E-I-H-D-R model in the main text. We here introduce the data and some other parameters in the model simulations process.

Table S1: The population, Log likelihood value and Log likelihood value standard derivation

Country	Population	Log likelihood value	Log likelihood value SD
India	1393409033	-963.1556	0.1627
Indonesia	276361788	-731.9173	0.1813
Malaysia	32776195	-553.4498	0.1103
Bangladesh	166303494	-577.8098	0.0946
Nepal	29674920	-447.7515	0.1041
Myanmar	54806014	-419.5027	0.0827

In Table S1, the population is the whole population in each country at the time when we started our study. We assume the population is constant during our study period. The Log likelihood value is the current optimal outcome of our codes. The Log likelihood value standard derivation is the standard derivation of the The Log likelihood value we computed ten times under the optimal condition.

Table S2: The parameters in the model simulation

	ϕ	θ	θ_1	α	t_1	τ	BS.0	BE.0	BI.0	BT.0	BD.0	BR.0
India	0.0185	0.4	0.05	0.2978	2021.97695	0.1654	0.99	6	6	1	0	0.01
Indonesia	0.0232	0.404	0.01	0.2393	2021.97221	0.0425	0.99	14	14	1	0	0.01
Malaysia	0.0423	0.4	0.05	0.1526	2022.04799	0.1459	0.99	12	12	1	0	0.01
Bangladesh	0.0125	0.4	0.24	0.1	2021.95745	0.1011	0.99	9	9	1	0	0.01
Nepal	0.0195	0.4	0.24	0.1	2021.90909	0.1647	0.99	3	3	0	0	0.01
Myanmar	0.0168	0.4	0.05	0.1	2021.92475	0.3595	0.99	6	6	1	0	0.01

In Table S2, ϕ is the estimated infection severity case ratio. θ is the first immune evasion proportion and θ_1 is the second immune evasion proportion. θ and θ_1 represent how much loss of immunity for the recovered (R) and vaccinated (V) at time t_1 and $t_1 + 120$ days, respectively. We presume the second invasion occurred at $t_1 + 120$ days for simplicity. α is the relative ratio of Omicron IFR vs pre-Omicron IFR. t_1 is the Omicron first invasion time in each country. For example, $t_1 = 2021.97695$ in India, this

*Corresponding author: daihai.he@polyu.edu.hk

means the Omicron first invasion time is on the day 0.97695×365, 2021. τ is the over dispersion, which appears in the $Z_{t+\Delta t} \sim Negative\_Binominal(mean = D_{t+\Delta t}, variance = D_{t+\Delta t}(1 + \tau D_{t+\Delta t}))$. Δt in this equation is the τ. BS.0, BE.0, BI.0, BT.0, BD.0 and BR.0 are the system state initial values.

In Table S3, we show the cubic spline nodes number and nodes value ($log.\beta_i$, $i = 1, \ldots, 16$) of the transmission rate $\beta(t)$ [1–5].

The data and parameters listed in Table S1-Table S3 as an example to show what kind of detailed information we need to get the results in the main text. Actually, one of the result (Scenario 4, Fig.S4) in the next section is generated using these data and parameters. There would be some minor changes or adjustments to the parameters when we want to set different assumptions and fit different scenarios.

Table S4 shows the ratio between excess mortality rate and reported COVID-19 mortality rate [6].

Table S3: The cubic spline nodes number and nodes value of the transmission rate $\beta(t)$ ($log.\beta_i$, $i = 1, \ldots, 16$).

Country	n_β	$log.\beta_1$	$log.\beta_2$	$log.\beta_3$	$log.\beta_4$	$log.\beta_5$	$log.\beta_6$	$log.\beta_7$	$log.\beta_8$
India	16	5.9	5.056	4.8214	4.7973	4.9078	4.7835	5.214	5.3882
Indonesia	16	5.8993	4.7829	4.7769	4.7093	4.764	4.8728	4.85	4.9861
Malaysia	16	5.6779	4.311	4.4027	5.1247	4.7031	4.731	4.6626	4.8984
Bangladesh	16	5.775	4.9891	4.8336	4.6924	4.9821	4.7943	5.1501	5.1045
Nepal	16	4.5535	5.3215	4.781	4.9321	4.7605	4.8068	4.6099	5.4652
Myanmar	16	5.5742	4.3581	2.6564	5.7855	4.6862	4.8636	4.1881	4.9389

Country	$log.\beta_9$	$log.\beta_{10}$	$log.\beta_{11}$	$log.\beta_{12}$	$log.\beta_{13}$	$log.\beta_{14}$	$log.\beta_{15}$	$log.\beta_{16}$
India	5.7451	6.0895	6.2625	6.1876	6.1875	6.8749	6.7429	6.1937
Indonesia	5.4297	6.4258	6.7963	6.2139	6.2138	6.1877	6.7386	6.2015
Malaysia	4.9958	5.65	6.4417	6.1876	6.1877	6.1882	6.8805	6.2543
Bangladesh	5.5265	6.5185	6.8807	6.4069	6.4075	6.8657	6.2533	6.54
Nepal	5.4071	6.2198	6.2433	6.1876	6.1876	6.188	6.1924	6.8255
Myanmar	5.5056	5.6104	6.7413	6.1877	6.1876	6.1878	6.2338	6.5149

Table S4: The ratio of excess death vs report deaths and its 95% CI.

Country	Ratio of excess death vs report deaths	95% CI of the ratio
India	8.33	[7.58, 8.92]
Indonesia	5.11	[4.12, 6.63]
Myanmar	5.25	[3.73, 7.26]
Malaysia	1.53	[1.28, 1.85]
Nepal	10.61	[9.22, 12.2]
Bangladesh	14.72	[12.36, 17.94]

S2 Literature Statistics

We selected six countries as our research objects. And, we think some of these countries are less studied in the previous studies. We want to show this statement via the specific statistics shown in Table S5. We chose the different phrase combining with different country names as the search keywords. Then we applied google scholar to search these keywords and to get the total literature numbers.

Here we set India as an example to explain this search process. 'Country name COVID-19 Mathematical Modeling (Anywhere in the article)' mow means that the search keywords are 'India COVID-19 Mathematical Modeling' with the search setting 'Anywhere in the article'. After this search, we find there are 46,100 papers include the keywords 'India COVID-19 Mathematical Modeling' anywhere in the articles.

From Table S5, we can see that Nepal, Malaysia, Bangladesh and Myanmar obviously are the less studied countries in the previous studies.

Table S5: Literature statistics under different keywords and search settings

Numbers / Country / Keywords	India	Nepal	Indonesia	Malaysia	Bangladesh	Myanmar
Country name COVID-19 Mathematical Modeling (Anywhere in the article)	46,100	8,690	24,200	22,400	22,200	4,410
Country name COVID-19 (In the title of the article)	10,800	974	13,600	2,040	2,680	234
Country name COVID-19 Modeling (In the title of the article)	96	5	54	12	23	1
Country name Omicron (In the title of the article)	39	2	16	5	6	4

S3 The results under different scenarios

To test the sensitivity and consistency of our model,we set the different assumptions and scenarios to fit the model. We list several of them as following:

- Scenario 1: Consider "a single" invasion scenario of Omicron variants, to fit the transmission rate $\beta(t)$ (in the unit of $R_0(t)$) with 17 cubic spline nodes, $\psi = 1/2$;
- Scenario 2: Consider "a single" invasion scenario of Omicron variants, to fit the transmission rate $\beta(t)$ (in the unit of $R_0(t)$) with 17 cubic spline nodes, $\psi = 1/3$;
- Scenario 3: Consider multiple (e.g. "twice") invasions scenario by initial Omicron BA1 (or BA2 or both) variant and follow-up Omicron (BA4 or BA5) variant,to fit the transmission rate $\beta(t)$ (in the unit of $R_0(t)$) with 17 cubic spline nodes, $\psi = 1/2$;
- Scenario 4: Consider multiple (e.g. "twice") invasions scenario by initial Omicron BA1 (or BA2 or both) variant and follow-up Omicron (BA4 or BA5) variant,to fit the transmission rate $\beta(t)$ (in the unit of $R_0(t)$) with 16 cubic spline nodes, $\psi = 1/3$.

S3.1 Scenario 1

In this subsection, we consider "a single" invasion scenario of Omicron variants, to fit the transmission rate $\beta(t)$ (in the unit of $R_0(t)$) with 17 cubic spline nodes, $\psi = 1/2$. The model simulation result is shown in Figure S1.

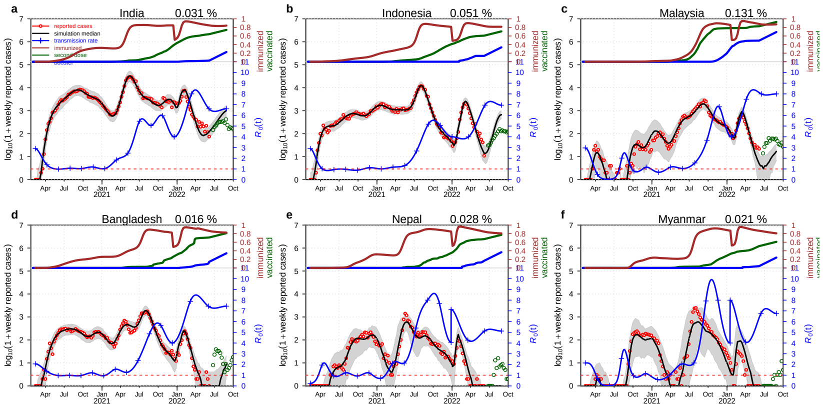

Figure S1: Model simulated death vs. reported death with the assumption of only one Omicron invasion and the rate of loss of immunity protection $\psi = 1/2$ in India(a), Indonesia(b), Malaysia(c), Bangladesh(d), Nepal(e) and Myanmar(f). The brown curve, green curve and blue curve at the top of each panel show the currently immunized people per capita, the number of persons who received a second dose per capita and the number of persons who received a booster dose per capita, respectively. The sudden drop in the brown curve shows the immune evasion caused by one Omicron variant invasion. The red circles, black curve and blue cure with the plus sign at the bottom of each panel show the reported cases, the simulation median and the transmission rate (in the unit of $R_0(t) = \beta(t)/\gamma$), respectively. The grey region denotes the 95% CI of 1000 model simulations. The green circles that overlap the black curve are the reported deaths for testing part. The extra green circles are the reported deaths to show the COVID-19 pandemic trends. The percent on the top of each panel is the estimated maximum log likelihood pre-Omicron IFR.

S3.2 Scenario 2

In this subsection, we consider "a single" invasion scenario of Omicron variants, to fit the transmission rate $\beta(t)$ (in the unit of $R_0(t)$) with 17 cubic spline nodes, $\psi = 1/3$. The model simulation result is shown in Figure S2.

S3.3 Scenario 3

In this subsection, we consider multiple (e.g. "twice") invasions scenario by initial Omicron BA1 (or BA2 or both) variant and follow-up Omicron (BA4 or BA5) variant,to fit the transmission rate $\beta(t)$ (in the unit of $R_0(t)$) with 17 cubic spline nodes, $\psi = 1/2$. The model simulation result is shown in Figure S3.

S3.4 Scenario 4

In this subsection, we consider multiple (e.g. "twice") invasions scenario by initial Omicron BA1 (or BA2 or both) variant and follow-up Omicron (BA4 or BA5) variant,to fit the transmission rate $\beta(t)$ (in the unit of $R_0(t)$) with 16 cubic spline nodes, $\psi = 1/3$. The model simulation result is shown in Figure S4.

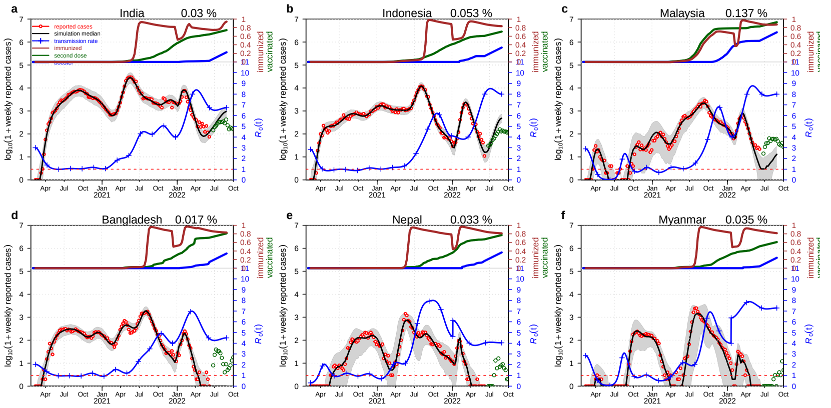

Figure S2: Model simulated death vs. reported death with the assumption of only one Omicron invasion and the rate of loss of immunity protection $\psi = 1/3$ in India(a), Indonesia(b), Malaysia(c), Bangladesh(d), Nepal(e) and Myanmar(f). The brown curve, green curve and blue curve at the top of each panel show the currently immunized people per capita, the number of persons who received a second dose per capita and the number of persons who received a booster dose per capita, respectively. The sudden drop in the brown curve shows the immune evasion caused by one Omicron variant invasion. The red circles, black curve and blue cure with the plus sign at the bottom of each panel show the reported cases, the simulation median and the transmission rate (in the unit of $R_0(t) = \beta(t)/\gamma$), respectively. The grey region denotes the 95% CI of 1000 model simulations. The green circles that overlap the black curve are the reported deaths for testing part. The extra green circles are the reported deaths to show the COVID-19 pandemic trends. The percent on the top of each panel is the estimated maximum log likelihood pre-Omicron IFR.

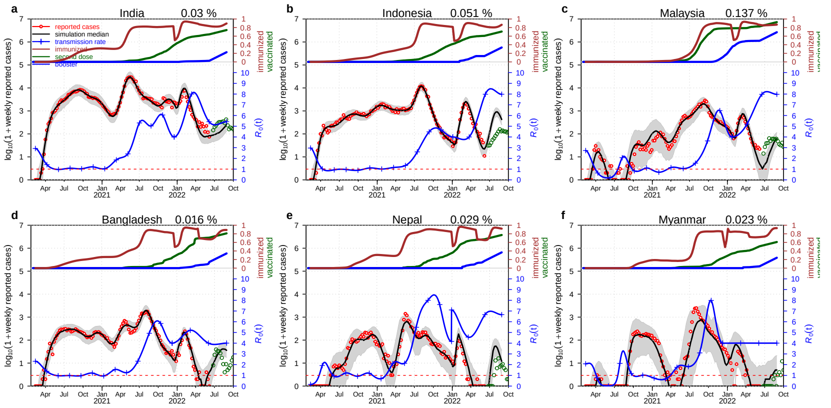

Figure S3: Model simulated death vs. reported death with the assumption of two Omicron invasions and the rate of loss of immunity protection $\psi = 1/2$ in India(a), Indonesia(b), Malaysia(c), Bangladesh(d), Nepal(e) and Myanmar(f). The brown curve, green curve and blue curve at the top of each panel show the currently immunized people per capita, the number of persons who received a second dose per capita and the number of persons who received a booster dose per capita, respectively. The sudden drops in the brown curve demonstrate the immune evasion caused by two Omicron variant invasions. The red circles, black curve and blue curve with the plus sign at the bottom of each panel show the reported cases, the simulation median and the transmission rate (in the unit of $R_0(t) = \beta(t)/\gamma$), respectively. The grey region denotes the 95% CI of 1000 model simulations. The green circles that overlap the black curve are the reported deaths for testing part. The extra green circles are the reported deaths to show the COVID-19 pandemic trends. The percent on the top of each panel is the estimated maximum log likelihood pre-Omicron IFR.

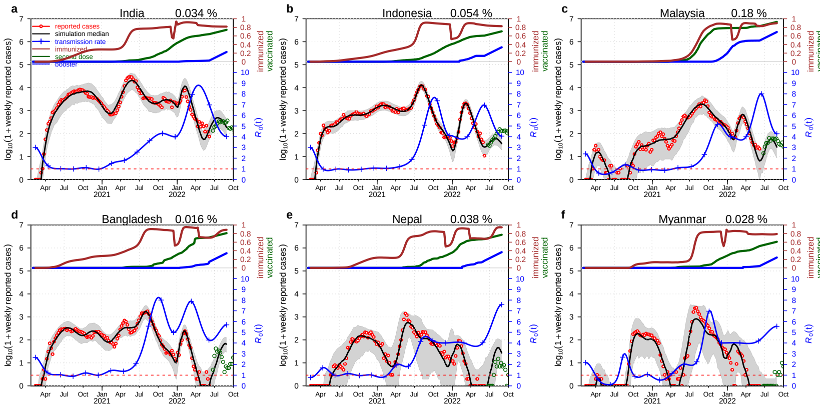

Figure S4: Model simulated death vs. reported death with the assumption of twice Omicron invasions and the rate of loss of immunity protection $\psi = 1/3$ in India(a), Indonesia(b), Malaysia(c), Bangladesh(d), Nepal(e) and Myanmar(f). The brown curve, green curve and blue curve at the top of each panel show the currently immunized people per capita, the number of persons who received a second dose per capita and the number of persons who received a booster dose per capita, respectively. The sudden drops in the brown curve demonstrate the immune evasion caused by two Omicron variant invasions. The red circles, black curve and blue curve with the plus sign at the bottom of each panel show the reported cases, the simulation median and the transmission rate (in the unit of $R_0(t) = \beta(t)/\gamma$), respectively. The grey region denotes the 95% CI of 1000 model simulations. The green circles that overlap the black curve are the reported deaths for testing part. The extra green circles are the reported deaths to show the COVID-19 pandemic trends. The percent on the top of each panel is the estimated maximum log likelihood pre-Omicron IFR.

References

[1] Richard H Bartels, John C Beatty, and Brian A Barsky. *An introduction to splines for use in computer graphics and geometric modeling.* Morgan Kaufmann, 1995.

[2] William T Vetterling et al. *Numerical recipes example book (c++): The art of scientific computing.* Cambridge University Press, 2002.

[3] Daihai He et al. "New estimates of the Zika virus epidemic attack rate in Northeastern Brazil from 2015 to 2016: A modelling analysis based on Guillain-Barré Syndrome (GBS) surveillance data". In: *PLoS neglected tropical diseases* 14.4 (2020), e0007502.

[4] Lewi Stone et al. "Extraordinary curtailment of massive typhus epidemic in the Warsaw Ghetto". In: *Science advances* 6.30 (2020), eabc0927.

[5] Shi Zhao et al. "Modelling the large-scale yellow fever outbreak in Luanda, Angola, and the impact of vaccination". In: *PLoS neglected tropical diseases* 12.1 (2018), e0006158.

[6] Haidong Wang et al. "Estimating excess mortality due to the COVID-19 pandemic: a systematic analysis of COVID-19-related mortality, 2020–21". In: *The Lancet* 399.10334 (2022), pp. 1513–1536.
